# Associations Between Arterial Oxygen Saturation, Body Size and Limb Measurements Among High-Altitude Andean Children

**DOI:** 10.1002/ajhb.22422

**Published:** 2013-08-01

**Authors:** Emma Pomeroy, Jay T Stock, Sanja Stanojevic, J Jaime Miranda, Tim J Cole, Jonathan CK Wells

**Affiliations:** 1 Division of Biological Anthropology, Department of Archaeology and Anthropology, University of CambridgeCambridge, United Kingdom; 2 Child Health Evaluative Sciences, The Hospital for Sick ChildrenToronto, Ontario, Canada; 3 CRONICAS Centre of Excellence in Chronic Diseases and Department of Medicine, School of Medicine, Universidad Peruna Cayetano HerediaLima, Peru; 4 MRC Centre of Epidemiology for Child Health, Institute of Child Health, University College LondonLondon, United Kingdom; 5 Childhood Nutrition Research Centre, Institute of Child Health, University College LondonLondon, United Kingdom

## Abstract

**Objectives:**

The relative influences of hypoxia and other environmental stressors on growth at altitude remain unclear. Previous work demonstrated an association between peripheral arterial oxygen saturation (S_p_O_2_) and anthropometry (especially tibia length) among Tibetan and Han children at altitude. We investigated whether similar associations exist among Andeans, and the patterning of associations between S_p_O_2_ and anthropometry.

**Methods:**

Stature, head-trunk height, total upper and lower limb lengths, zeugopod (ulna and tibia) and autopod (hand and foot) lengths were measured in Peruvian children (0.5**–**14 years) living at >3000 m altitude. S_p_O_2_ was measured by pulse oximetry. Anthropometry was converted to internal *z* scores. Correlation and multiple regression were used to examine associations between anthropometry *z* scores and S_p_O_2_, altitude, or S_p_O_2_ adjusted for altitude since altitude is a major determinant of variation in S_p_O_2_.

**Results:**

S_p_O_2_ and altitude show weak, significant correlations with zeugopod length *z* scores and still weaker significant correlations with total upper and lower limb length *z* scores. Correlations with *z* scores for stature, head-trunk height**,** or autopod lengths are not significant. Adjusted for altitude, there is no significant association between anthropometry and S_p_O_2_.

**Conclusions:**

Associations between S_p_O_2_ or altitude and total limb and zeugopod length *z* scores exist among Andean children. However, the relationships are relatively weak, and while the relationship between anthropometry and altitude may be partly mediated by S_p_O_2,_ other factors that covary with altitude (e.g., socioeconomic status, health) are likely to influence anthropometry. The results support suggestions that zeugopod lengths are particularly sensitive to environmental stressors. Am. J. Hum. Biol., 25:629–636, 2013. © 2013 Wiley Periodicals, Inc.

As an environmental stressor that cannot be culturally mitigated (Baker, 1976), high altitude hypoxia has been the subject of considerable research to identify the genetic, physiological, and morphological means by which humans adapt to these conditions. Whether hypoxia directly affects human growth has been extensively investigated, and the negative impact of high altitude pregnancy on birth weight is well documented (Beall, 1981; Giussani et al., 2001; Haas et al., 1977; McClung, 1969; Moore et al., 1998; Unger et al., 1988; Zamudio and Moore, 2000). While a number of studies report reduced child and adult stature at altitude, the growing consensus is that socioeconomic differences account for the greatest part of the deficit, while a reduction of just 1–2 cm in adult stature is likely attributable to hypoxia (Greksa, 2006). This is important because growth deficits due to the direct effects of hypoxia may be difficult to resolve, while deficits due to other factors can be effectively addressed by interventions to improve growth.

The delivery of oxygen to the tissues is not only critical for maintaining immediate function, but also for growth. This is demonstrated by the fact that populations who have lived at altitude for many generations (e.g., Andeans, Tibetans) are partially protected from hypoxia-related fetal growth reduction by genetic adaptations that increase oxygen delivery to the fetus (Bennett et al., 2008; Giussani et al., 2001; Julian et al., 2007,^2009^,^2011^; Moore, 2003). The body employs various mechanisms to counteract ambient hypoxia at high altitude and thus maintain cellular and tissue oxygen homeostasis (reviewed in Beall, 2001; Moore et al., 1998; West et al., 2007). These include erythrocytosis and increased ventilation and heart rates on acute exposure to hypoxia, and in the case of Tibetan high altitude natives, increased tissue blood flow (perfusion: Andeans remain untested in this respect) (Beall et al., 2001; Erzurum et al., 2007).

There is evidence that exposure to hypoxia *in utero* or post-natally affects body size and proportions, particularly the relative size of the limbs and trunk (Bailey et al., 2007; Lampl et al., 2003; Stinson 2009), although the extent to which hypoxia exerts a direct influence and the mechanisms by which it does so remain unclear. Recently the specific impact of oxygen delivery on human postnatal growth at altitude has been investigated using peripheral arterial oxygen saturation (S_p_O_2_). S_p_O_2_ measures the percentage of hem groups in hemoglobin which are bound to oxygen (Moore et al., 1998), and influences tissue oxygen delivery. S_p_O_2_ decreases with increasing altitude, even among populations adapted to high altitude hypoxia (Beall, 2007). S_p_O_2_ represents only one component of blood oxygen content (the total amount of oxygen the blood carries) which is also a function of erythrocyte and hemoglobin concentrations (Beall, 2001,^2007^). The advantage of S_p_O_2_ is that it can be measured simply, inexpensively and non-invasively by pulse oximetry (Schult and Canelo-Aybar, 2011), and evidence for a major gene among Tibetans that increases oxygen saturation (Beall et al., 1994,^1997^) demonstrates that S_p_O_2_ is sufficiently important to have been under natural selection in this native high altitude population. However, Andeans do not seem to share this adaptation (Beall et al., 2004).

Bailey and co-workers (Bailey and Hu, 2002; Bailey et al., 2007) demonstrated that higher S_p_O_2_ and better lung function are associated with greater stature and with relatively longer tibiae among 8- to 11-year-old Tibetan and Han children at high altitude. A similar relationship between anthropometry and S_p_O_2_ has not yet been observed in highland Andeans. The purpose of this study is to investigate the relationship between S_p_O_2_ and measures of body size, limb, and trunk lengths; specifically stature, head-trunk (sitting) height, total limb lengths and limb segment lengths (zeugopod: ulna or tibia; and autopod: hand or foot) among Peruvian infants and children at high altitude (>3000 m). While relative total lower limb length, and sometimes tibia length, have been investigated in studies of body proportions in relation to environmental stress, relationships between total upper limb length, ulna length, or autopod (hand or foot) length and S_p_O_2_ have not. The investigation of total upper and lower limb lengths, as well as limb segment lengths, may help to elucidate the mechanisms underlying altered body proportions under stress conditions (Pomeroy et al., 2012). These mechanisms remain unclear, but are relevant to understanding reported associations between early life conditions, body proportions, and chronic disease risk (reviewed in Bogin and Varela-Silva, 2010; Samaras, 2007) and how humans adapt to poor environmental conditions during growth. We hypothesize that, as among Tibetan and Han children, S_p_O_2_ in Andeans will be more strongly positively associated with zeugopod lengths than trunk length or stature. Furthermore, in light of evidence that the effects of environmental stress on anthropometry rank as follows: zeugopod > total limb > autopod > trunk (Pomeroy et al., 2012), we hypothesize that a similar ranking will be seen with S_p_O_2_.

## Methods

**Figure 1:**
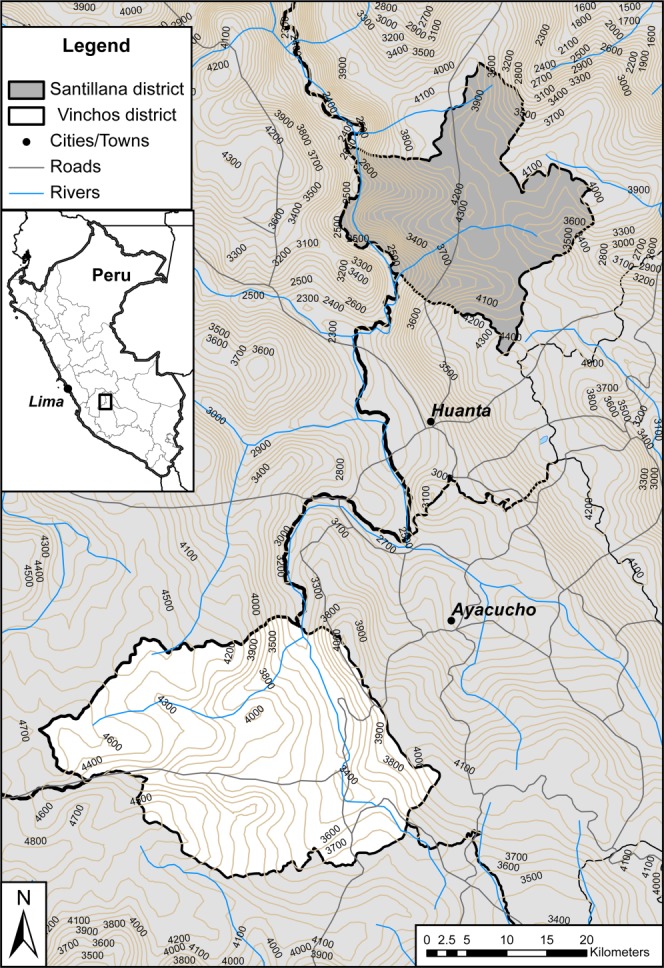
Map showing location of study sites in the central Peruvian highlands. [Color figure can be viewed in the online issue, which is available at wileyonlinelibrary.com.]

The study received ethical approval from the Institutional Ethics Committee at the Universidad Peruana Cayetano Heredia, Lima, and from the Health Directorate for Ayacucho Region (Dirección Régional de Salud Ayacucho, DIRESA). Participation was voluntary and the study was conducted according to international ethical standards (World Medical Association, 2008). Children aged 6 months to 14 years who were born and raised in highland communities in Ayacucho Region, Peru (Fig. 1), were included in the study. First language was predominantly Quechua, and participants came from small rural communities living from subsistence agriculture and herding at altitudes from 3100–4400 m. Written informed consent was obtained from a parent or legal guardian by signature or fingerprint (where not literate) once the study had been explained in full to them and to the participant in age-appropriate terms. Participants aged 6 years or over also gave their assent, either in writing or verbally where not literate.

Anthropometry was measured by a single trained observer (EP) using standard methods as previously described (Pomeroy et al., 2012). Measurements were converted to age–sex-specific *z* scores based on a combined sample of highland and lowland children who participated in a larger study (Pomeroy et al., 2012), though only highland children are considered here. *Z* scores adjust a measurement for age and sex and express it in standard deviation units. Therefore their use permits analyses combining data varying by age and sex so as to maximize statistical power. *Z* scores were derived by the LMS method (Cole, 1990; Cole and Green, 1992) using LMS Chartmaker Light version 2.43 (Pan and Cole, 2010). Subsequently, references to any anthropometric measurements are to their *z* scores.

S_p_O_2_ was measured by pulse oximetry, which uses differences in the wavelength of light absorbed by oxygenated and deoxygenated blood to estimate arterial oxygen saturation (S_a_O_2_). As deoxyhemoglobin absorbs more red light (600–750 nm wavelength) than oxyhemoglobin, which has higher infrared absorption (850–1000 nm), the ratio of light absorption in the red and infrared spectra indicates S_a_O_2_ (Fouzas et al., 2011). S_p_O_2_ is typically measured using a fingertip clip that passes red and infrared light through the finger and measures light transmission (Fouzas et al., 2011).

A Nonin 8500 pulse oximeter (Nonin Medical, Plymouth, MN) was used to measure S_p_O_2_. Probes were selected based on the participant’s weight according to the manufacturer’s recommendations, and attached to the index finger of the left hand, or to the big toe of infants whose fingertip thickness was less than 5 mm. Individuals were measured in a calm, resting state (if they were visibly distressed the measurement was not done). As movement can reduce measurement accuracy (Fouzas et al., 2011), the child’s hand or foot was held still during the measurement where necessary. Insufficient tissue perfusion can also lead to inaccurate readings (Fouzas et al., 2011), but results were only recorded when the oximeter’s heart rate indicator showed that there was sufficient blood flow for a reliable measurement. The manufacturer reports accuracy of ±2%, consistent with that for pulse oximetry in general (Jensen et al., 1998; Ross and Helms, 1990), and the 8500 model is approved for use up to 12,000 m altitude and is certified for aeromedical use by the US Air Force.

The normality of distributions for S_p_O_2_ and the anthropometric data were assessed visually using histograms. Anthropometry *z* score distributions were normal after removing a single strong outlier, and similarly for S_p_O_2_ after excluding two individuals with unusually low readings. Despite previous studies reporting an association between S_p_O_2_ and age (Schult and Canelo-Aybar, 2011), no such relationship was found in our data (Pearson’s correlation, *P* = 0.1). Sex differences in S_p_O_2_ were also absent (*t*-test, *P* = 0.3). Thus age and sex were not included in the analyses, as the anthropometry *z* scores were already age–sex-adjusted.

**Figure 2:**
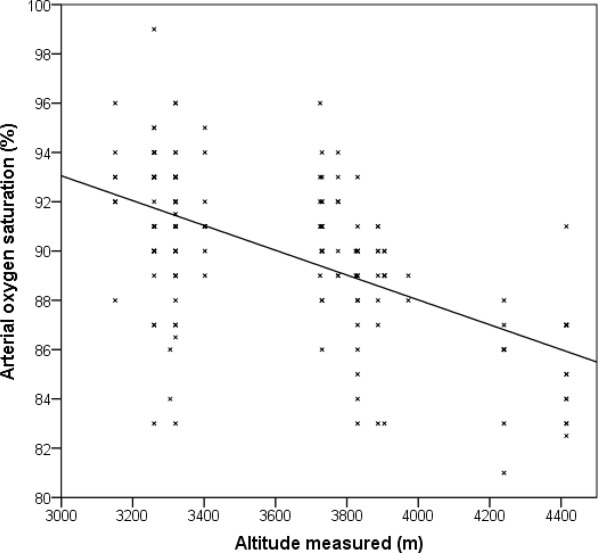
Scatter plot of peripheral arterial oxygen saturation (S_p_O_2_) against altitude where measurements were taken, demonstrating the expected decrease in S_p_O_2_ with increasing altitude.

The altitude at which the children were studied varied, and S_p_O_2_ decreases with increasing altitude. The sample fell into three natural groups in terms of altitude (Fig. 2), so the data were analyzed in three altitude groups and the correlations between altitude and S_p_O_2_ assessed using Spearman’s rank correlation coefficient. Pearson’s correlation was derived between S_p_O_2_ and anthropometry. As other factors varying with altitude, such as socioeconomic status (SES) and healthcare (see “Introduction” section), might also impact on body size and proportions, correlations between altitude of measurement and anthropometry *z* scores were also conducted to investigate whether the relationships were similar to those between anthropometry and S_p_O_2_, and thus whether a direct effect of S_p_O_2_, rather than other factors associated with altitude, might be inferred. Therefore, Spearman’s correlations were derived between altitude group and anthropometry *z* scores.

Multiple regression analyses of anthropometry *z* scores on S_p_O_2_ were used to assess their associations including an adjustment for altitude group. To confirm that the use of *z* scores did not bias the results, the analyses were repeated using raw anthropometry as outcomes, adjusting for sex, age and age^2^ (to cater for nonlinear relationships). Analyses were performed using SPSS 21.0 for Windows, with statistical significance defined at *P* < 0.05.

## Results

Table 1 summarizes the characteristics of the study sample. Summary statistics on stature, sitting height, and weight by age group and sex are given in Supporting Information Table S1, and further summary anthropometry can be found in Pomeroy et al. (2012).

**Table 1:** Summary statistics of the study sample

Variable	Statistic	Value
Sample size	*n* (males, females)	165 (82, 83)
Altitude of measurement (*m*)	Median	3564
	Interquartile range	3306–3823
	Range	3150–4415
S_p_O_2_ (%)	Median	90
	Interquartile range	88–92
	Range	81–99
Age (years)	Mean	5.25
	Standard deviation	3.59
	Range	0.5–14.4

The correlation between altitude of measurement and S_p_O_2_ (Fig. 2) is negative, as expected (*r* = −0.55, *P* < 0.001). Correlations between anthropometry and S_p_O_2_ (Table 2) are significant for ulna, tibia, total upper limb and total lower limb length *z* scores, but not for stature, head-trunk height, foot, or hand length *z* scores. Overall the correlations are low (*r* ≤0.23), indicating weak relationships even where significant.

**Table 2:** Correlations between S_p_O_2_ and anthropometry z scores

Outcome *z* score	Pearson *r*	*P*	*n*
Ulna length	0.23	**0.003**	163
Tibia length	0.22	**0.004**	164
Total upper limb length	0.19	**0.02**	159
Total lower limb length	0.17	**0.03**	165
Stature	0.15	0.06	165
Head-trunk height	0.08	0.3	165
Foot length	0.07	0.4	164
Hand length	0.05	0.5	156

Bold indicates significant *P* values.

Correlations between altitude of measurement and anthropometry *z* scores rank similarly to those for S_p_O_2_, with those for zeugopod length highest, and those for autopod lengths and head-trunk height low and insignificant (Table 3). Furthermore, these correlations with altitude are generally similar in magnitude to those with S_p_O_2_ (Table 2). However, none of the anthropometry *z* scores are significantly related to S_p_O_2_ when adjusted for altitude group (Table 4). The results are confirmed by repeating the analyses using raw anthropometry, which leads to the same conclusions (Supporting Information Tables S2–S4).

**Table 3:** Correlations between altitude and anthropometry z scores

Outcome*z* score	Spearman*r*	*P*	*n*
Tibia length	−0.28	**<0.001**	164
Ulna length	−0.26	**0.001**	163
Total lower limb length	−0.3	**0.001**	165
Total upper limb length	−0.18	**0.03**	159
Stature	−0.1	0.07	165
Hand length	−0.08	0.3	156
Foot length	−0.04	0.6	164
Head-trunk height	0.006	0.9	165

Bold indicates significant *P* values.

**Table 4:** Results of multiple regression models of anthropometry z scores on S_p_O_2_ and altitude

	3700–4000 m altitude^a^			4200–4500 m altitude			S_p_O_2_		
Outcome *z* score	B	SE	*P*	B	SE	*P*	B	SE	*P*
Tibia length	−0.32	0.11	**0.004**	−0.67	0.19	**0.001**	0.007	0.018	0.7
Total lower limb length	−0.37	0.12	**0.004**	−0.71	0.22	**0.002**	−0.002	0.021	0.9
Ulna length	−0.24	0.10	**0.02**	−0.43	0.18	**0.02**	0.018	0.017	0.3
Total upper limb length	−0.22	0.11	**0.05**	−0.12	0.19	0.5	0.027	0.018	0.1
Stature	−0.13	0.12	0.3	−0.42	0.22	0.06	0.009	0.021	0.6
Foot length	−0.00	0.13	0.9	−0.20	0.22	0.4	0.004	0.021	0.8
Hand length	−0.02	0.13	0.9	−0.04	0.22	0.8	0.009	0.021	0.7
Head-trunk height	0.13	0.13	0.3	0.04	0.23	0.8	0.021	0.022	0.3

Bold indicates significant *P* values.

a Two dummy variables for altitude group with lowest altitude group (3150–3400 m) as the reference group.

## Discussion

Oxygen saturation and anthropometry *z* scores are significantly associated in this population of high altitude Andean children. However, anthropometry *z* scores correlate similarly with altitude and S_p_O_2_, and adjusting for altitude the S_p_O_2_—anthropometry associations vanish. This suggests that the relationship between anthropometry and altitude may be partly mediated through S_p_O_2_, which itself is negatively correlated with altitude, but that other factors covarying with altitude also independently influence anthropometry. The low correlations between S_p_O_2_ and anthropometry indicate that, even if the association is causal, factors other than S_p_O_2_ substantially influence anthropometry, in line with evidence that nutritional and other factors, rather than hypoxia, most likely explain the height deficit in highland populations (Greksa, 2006).

The results are consistent with previous evidence that total lower limb length is more sensitive to the effects of environmental stress than trunk length (Bogin and Varela-Silva, 2010; Frisancho, 2007; Gunnell et al., 1998; Whitley et al., 2008), and that zeugopod lengths are more sensitive to environmental stress than total limb lengths (Meadows Jantz and Jantz, 1999; Pomeroy et al., 2012). With specific reference to hypoxia, the results agree with those of Bailey and colleagues on Tibetan and Han children, which also showed a stronger association between S_p_O_2_ and absolute or relative tibia length than with total lower limb length or stature (Bailey et al., 2007). They are also consistent with a study into the effect of prenatal hypoxia on rats demonstrating reduced total fore- and hind-limb lengths, and that zeugopod lengths were more strongly affected than stylopod (humerus or femur) lengths (Hunter and Clegg, 1973). However that study also reported significant reductions in paw length under hypoxic conditions which contrast with our results. Work examining relative tibia, femur, and total upper limb lengths in hypoxia-exposed fetuses also suggests that the tibia was shortened relative to the femur, but total upper limb length was unaffected (Lampl et al., 2003), again in contrast with our results.

The results suggest that factors other than S_p_O_2_ that covary with altitude may explain much of the altitude-related anthropometric variation. As already outlined, S_p_O_2_ is only one aspect of tissue oxygen delivery, and variation in altitude may have additional hypoxia-related effects on growth that are not captured by S_p_O_2_. S_p_O_2_ also varies diurnally in individuals, for example tending to be lower during sleep (Fouzas et al., 2011) and exercise (Brutsaert et al., 2000), so the single measurement of S_p_O_2_ recorded in this study may not have captured between-individual variation in S_p_O_2_ that may have had significant impacts on growth. Furthermore, environmental characteristics like SES, temperature and healthcare access decrease with increasing altitude (Leonard et al., 1990; Niermeyer et al., 2009; Rivera-Ch et al., 2008; West et al., 2007) while respiratory infection rates increase (Niermeyer et al., 2009; Subhi et al., 2009), and these may also impact on growth, body size, and body proportions.

Distinguishing between the influences of these different environmental stressors is challenging, since factors such as SES, ambient temperature, and tissue oxygenation (the end product of various mechanisms of oxygen delivery at altitude) are hard to characterize. In this study, participants came from small rural communities where SES is very low and varies little, while diet and access to healthcare are similarly poor. Bailey et al. (2007) reported little variation in SES in their sample of Tibetan and Han children, so argued this could not have accounted for their results, but it remains untested whether even such limited variation could still influence morphology at altitude. Variation in other factors known to influence growth and body size, including maternal phenotype, health and nutrition, and intergenerational effects on maternal and offspring phenotype (Wells, 2010) are important considerations for future research. Temperature is unlikely to explain the results, which are inconsistent with the mechanisms thought to link limb growth and temperature (Pomeroy et al., 2012). Given the difficulty of separating different influences on morphology at altitude where exposure to multiple stressors is correlated, experimental animal models where other factors can be controlled are likely key to understanding the effects of hypoxia on body size and proportions.

In terms of underlying mechanisms, our results are inconsistent with a proximo-distal decrease in available resources along the limb, as suggested in Lampl’s distal blood flow model (Lampl et al., 2003). This hypothesis states that fetuses exposed to hypoxia due to maternal diabetes or smoking show reduced tibia length, but not reduced total upper limb or femur lengths, due to the nature of the fetal circulation and the diminution of blood oxygen availability with distance from the placenta, reaching its lowest levels in the distal lower limb (tibia). The model implies that hands and feet are most affected by hypoxia, though this was not investigated by Lampl et al. (2003). However, this was not the pattern observed here.

The results are more consistent with the thrifty phenotype hypothesis as applied to limb lengths (Pomeroy et al., 2012). The thrifty phenotype hypothesis (Hales and Barker, 1992) states that when resources are limited, growth is prioritized in organs or parts of the body where function would be most compromised by inadequate growth. In comparing children from the Peruvian highlands (including the sample from this study) with those from the Peruvian lowlands who experience markedly lower levels of environmental stress in terms of socioeconomic factors, healthcare access, hypoxia and cold exposure, we documented a similar pattern (Pomeroy et al., 2012). Zeugopod lengths showed the greatest differences between populations, followed by total limb lengths, while differences were smaller in hand and foot lengths and smallest in head-trunk height. The trunk may be relatively protected as it houses the major organs, while autopod lengths may also be protected due to their critical roles in manipulation and substrate interaction during locomotion. While this suggestion has not been demonstrated empirically, it has been proposed that greater canalization of autopod size compared with the stylopod and zeugopod may be explained in this way (Rolian, 2008,^2009^; Young and Hallgrímsson 2005). Our analyses suggest that S_p_O_2_ represents one altitude-associated signal of supply, to which the limb components respond in a hierarchical manner.

In terms of proximate mechanisms, trunk size may be maintained at the expense of limb lengths through peripheral vasoconstriction which may reduce nutrient delivery to the limbs, a response observed in humans and animal models exposed to hypoxia or nutritional stress (Burrage et al., 2009; Celander, 1960; Edelstone and Rudolph, 1979; Gardner et al., 2002; Giussani et al., 1994,^2005^; Hawkins et al., 2000; Kidd et al., 1966; Krampl et al., 2001; Llanos et al., 2007; Morrison, 2008; Mulder et al., 1998; Powers and Swyer, 1977; Rudolph, 1984; Williams et al., 2005). Existing studies of regional blood flow generally measure only lower limb blood supply, and data on upper limb circulation are lacking, as are direct studies of blood flow in relation to limb segment lengths in animals or humans. Studies of limb segment blood flow demonstrate proportionally greater flow (corrected for element size) to the stylopod than the zeugopod, although there is evidence for variation with age (proximo-distal gradients decrease with age) and among species (Morris and Kelly, 1980; Nakano et al., 1986; Tothill et al., 1985; Tothill and MacPherson, 1986). In addition, it is well accepted that blood supply affects limb growth (Brashear, 1963; Brodin, 1955; Serrat, 2007; Tomita et al., 1986).

A strength of this study is the predominance of infants and young children in the sample, since plasticity is thought to be greatest at younger ages (Lucas, 1991; Mei et al., 2004; Smith et al., 1976) and thus patterns of body size and proportion in relation to S_p_O_2_ are potentially strongest in such individuals. While fetal growth may be the most plastic of all, prenatal environmental influences are mediated through maternal phenotype (Wells, 2003). Limitations of this study include its short timescale, so it was not possible to test whether the patterns maintain into adulthood, or to investigate the interaction between hypoxia and nutritional status on growth suggested by Bailey et al. (2007). It also remains to be demonstrated how other aspects of oxygen delivery relate to body proportions and growth at altitude. Nonetheless the study has important implications for public health policy. As correlations between S_p_O_2_ and anthropometry were low, even where significant, this implies that other factors exert much greater influences on growth and variation in body size and proportions, likely nutrition and healthcare as others have argued previously (e.g., Greksa, 2006). Therefore, interventions that serve to improve nutrition and health in highland communities are likely to be effective in improving growth.

In conclusion, this study demonstrates significant correlations between S_p_O_2_ or altitude and limb and limb segment length *z* scores among Andean children. The results indicate that associations are strongest with zeugopod length and then total limb length *z* scores, but weaker and insignificant with head-trunk height and autopod length *z* scores. Part of the association between altitude and limb measurement *z* scores seems to be mediated by S_p_O_2_, although correlations are relatively weak, and other factors that covary with altitude are likely to play a major role in influencing body size and proportions. Future work should aim to explore further the effects of hypoxia on growth and body proportions to elucidate the details of the underlying mechanisms, and to distinguish the effects of hypoxia and other environmental stress exposures at altitude.
