# Supplementary material for: Associations Between Arterial Oxygen Saturation, Body Size and Limb Measurements Among High-Altitude Andean Children
Source: Am J Hum Biol. 2013 Aug 1;25(5):629–36. doi: 10.1002/ajhb.22422 (PMC3793237; doi:10.1002/ajhb.22422)
Supplement: Supplementary file 1 [file ajhb0025-0629-sd1.doc]

Title: Associations between arterial oxygen saturation, body size and limb measurements among high-altitude Andean children

**Authors:** Emma Pomeroy ^a^

Jay T. Stock ^a^

Sanja Stanojevic ^b^

J. Jaime Miranda ^c^

Tim J. Cole ^d^

Jonathan C.K. Wells ^e^

Institutions:

^a^ Division of Biological Anthropology, Department of Archaeology and Anthropology, University of Cambridge, UK

^b^ Child Health Evaluative Sciences, The Hospital for Sick Children, Toronto, Ontario, Canada

^c^ CRONICAS Centre of Excellence in Chronic Diseases and Department of Medicine, School of Medicine, Universidad Peruana Cayetano Heredia, Lima, Peru

^d^ MRC Centre of Epidemiology for Child Health, Institute of Child Health, University College London, UK

^e^ Childhood Nutrition Research Centre, Institute of Child Health, University College London, UK

Short title: Arterial oxygen saturation and limb proportions

Corresponding author details: Dr Emma Pomeroy

Newnham College

Cambridge

CB3 9DF

UK

Tel: +44 7815 189784

Email: emma.pomeroy@cantab.net

Funding: EP received PhD funding from the Arts and Humanities Research Council (UK), and fieldwork grants from the University of Cambridge Centre for Latin American Studies Abbey-Santander Travel Grants. She is currently supported by a Junior Research Fellowship from Newnham College, Cambridge. JJM is with the CRONICAS Center of Excellence in Chronic Diseases at the Universidad Peruana Cayetano Heredia which is funded by the National Heart, Lung and Blood Institute, National Institutes of Health, Department of Health and Human Services, under contract No. HHSN268200900033C. TJC is funded by Medical Research Council grant MR/J004839/1.

Abstract

Objectives: The relative influences of hypoxia and other environmental stressors on growth at altitude remain unclear. Previous work demonstrated an association between peripheral arterial oxygen saturation (S_p_O_2_) and anthropometry (especially tibia length) among Tibetan and Han children at altitude. We investigated whether similar associations exist among Andeans, and the patterning of associations between S_p_O_2_ and anthropometry.

Methods: Stature, head-trunk height, total upper and lower limb lengths, zeugopod (ulna and tibia) and autopod (hand and foot) lengths were measured in Peruvian children (0.5-14 years) living at >3000m altitude. S_p_O_2_ was measured by pulse oximetry. Anthropometry was converted to internal z scores. Correlation and multiple regression were used to examine associations between anthropometry z scores and S_p_O_2_, altitude, or S_p_O_2_ adjusted for altitude since altitude is a major determinant of variation in S_p_O_2_.

Results: S_p_O_2_ and altitude show weak, significant correlations with zeugopod length z scores and still weaker significant correlations with total upper and lower limb length z scores. Correlations with z scores for stature, head-trunk height or autopod lengths are not significant. Adjusted for altitude, there is no significant association between anthropometry and S_p_O_2_.

Conclusions: Associations between S_p_O_2_ or altitude and total limb and zeugopod length z scores exist among Andean children. However, the relationships are relatively weak, and while the relationship between anthropometry and altitude may be partly mediated by S_p_O_2_, other factors that covary with altitude (e.g. socioeconomic status, health) are likely to influence anthropometry. The results support suggestions that zeugopod lengths are particularly sensitive to environmental stressors.

Key words: arterial oxygen saturation, limb lengths, trunk length, altitude

Introduction

As an environmental stressor that cannot be culturally mitigated (Baker 1976), high altitude hypoxia has been the subject of considerable research to identify the genetic, physiological and morphological means by which humans adapt to these conditions. Whether hypoxia directly affects human growth has been extensively investigated, and the negative impact of high altitude pregnancy on birth weight is well documented (Beall 1981; Giussani et al 2001; Haas et al 1977; McClung 1969; Moore et al 1998; Unger et al 1988; Zamudio and Moore 2000). While a number of studies report reduced child and adult stature at altitude, the growing consensus is that socioeconomic differences account for the greatest part of the deficit, while a reduction of just 1-2cm in adult stature is likely attributable to hypoxia (Greksa 2006). This is important because growth deficits due to the direct effects of hypoxia may be difficult to resolve, while deficits due to other factors can be effectively addressed by interventions to improve growth.

The delivery of oxygen to the tissues is not only critical for maintaining immediate function, but also for growth. This is demonstrated by the fact that populations who have lived at altitude for many generations (e.g. Andeans, Tibetans) are partially protected from hypoxia-related foetal growth reduction by genetic adaptations that increase oxygen delivery to the foetus (Bennett et al 2008; Giussani et al 2001; Julian et al 2011; Julian et al 2007; Julian et al 2009; Moore 2003). The body employs various mechanisms to counteract ambient hypoxia at high altitude and thus maintain cellular and tissue oxygen homeostasis (reviewed in Beall 2001; Moore et al 1998; West et al 2007). These include erythrocytosis and increased ventilation and heart rates on acute exposure to hypoxia, and in the case of Tibetan high altitude natives, increased tissue blood flow (perfusion: Andeans remain untested in this respect) (Beall et al 2001; Erzurum et al 2007).

There is evidence that exposure to hypoxia *in utero* or postnatally affects body size and proportions, particularly the relative size of the limbs and trunk (Bailey et al 2007; Lampl et al 2003; Stinson 2009), although the extent to which hypoxia exerts a direct influence and the mechanisms by which it does so remain unclear. Recently the specific impact of oxygen delivery on human postnatal growth at altitude has been investigated using peripheral arterial oxygen saturation (S_p_O_2_). S_p_O_2_ measures the percentage of haem groups in haemoglobin which are bound to oxygen (Moore et al 1998), and influences tissue oxygen delivery. S_p_O_2_ decreases with increasing altitude, even among populations adapted to high altitude hypoxia (Beall 2007). S_p_O_2_ represents only one component of blood oxygen content (the total amount of oxygen the blood carries) which is also a function of erythrocyte and haemoglobin concentrations (Beall 2001; 2007). The advantage of S_p_O_2_ is that it can be measured simply, inexpensively and non-invasively by pulse oximetry (Schult and Canelo-Aybar 2011), and evidence for a major gene among Tibetans that increases oxygen saturation (Beall et al 1994; Beall et al 1997) demonstrates that S_p_O_2_ is sufficiently important to have been under natural selection in this native high altitude population, However, Andeans do not seem to share this adaptation (Beall et al 2004).

Bailey and co-workers (Bailey and Hu 2002; Bailey et al. 2007) demonstrated that higher S_p_O_2_ and better lung function are associated with greater stature and with relatively longer tibiae among 8 to 11 year-old Tibetan and Han children at high altitude. A similar relationship between anthropometry and S_p_O_2_ has not yet been observed in highland Andeans. The purpose of this study is to investigate the relationship between S_p_O_2_ and measures of body size, limb and trunk lengths; specifically stature, head-trunk (sitting) height, total limb lengths and limb segment lengths (zeugopod: ulna or tibia; and autopod: hand or foot) among Peruvian infants and children at high altitude (>3,000m). While relative total lower limb length, and sometimes tibia length, have been investigated in studies of body proportions in relation to environmental stress, relationships between total upper limb length, ulna length or autopod (hand or foot) length and S_p_O_2_ have not. The investigation of total upper and lower limb lengths, as well as limb segment lengths, may help to elucidate the mechanisms underlying altered body proportions under stress conditions (Pomeroy et al 2012). These mechanisms remain unclear, but are relevant to understanding reported associations between early life conditions, body proportions, and chronic disease risk (reviewed in Bogin and Varela-Silva 2010; Samaras 2007) and how humans adapt to poor environmental conditions during growth. We hypothesise that, as among Tibetan and Han children, S_p_O_2_ in Andeans will be more strongly positively associated with zeugopod lengths than trunk length or stature. Furthermore, in light of evidence that the effects of environmental stress effects on anthropometry rank as follows: zeugopod > total limb > autopod > trunk (Pomeroy et al 2012), we hypothesise that a similar ranking will be seen with S_p_O_2_.

Methods

The study received ethical approval from the Institutional Ethics Committee at the Universidad Peruana Cayetano Heredia, Lima, and from the Health Directorate for Ayacucho Region (Dirección Régional de Salud Ayacucho, DIRESA). Participation was voluntary and the study was conducted according to international ethical standards (World Medical Association 2008). Children aged 6 months to 14 years who were born and raised in highland communities in Ayacucho Region, Peru (Fig. 1), were included in the study. First language was predominantly Quechua, and participants came from small rural communities living from subsistence agriculture and herding at altitudes from 3,100-4,400m. Written informed consent was obtained from a parent or legal guardian by signature or fingerprint (where not literate) once the study had been explained in full to them and to the participant in age-appropriate terms. Participants aged 6 years or over also gave their assent, either in writing or verbally where not literate.

*** INSERT FIGURE 1 HERE ***

Anthropometry was measured by a single trained observer (EP) using standard methods as previously described (Pomeroy et al 2012). Measurements were converted to age-sex-specific z scores based on a combined sample of highland and lowland children who participated in a larger study (Pomeroy et al 2012), though only highland children are considered here. Z scores adjust a measurement for age and sex and express it in standard deviation units. Therefore their use permits analyses combining data varying by age and sex so as to maximise statistical power. Z scores were derived by the LMS method (Cole 1990; Cole and Green 1992) using LMS Chartmaker Light version 2.43 (Pan and Cole 2010). Subsequently, references to any anthropometric measurements are to their z scores.

S_p_O_2_ was measured by pulse oximetry, which uses differences in the wavelength of light absorbed by oxygenated and deoxygenated blood to estimate arterial oxygen saturation (S_a_O_2_). As deoxyhaemoglobin absorbs more red light (600-750nm wavelength) than oxyhaemoglobin, which has higher infrared absorption (850-1000nm), the ratio of light absorption in the red and infrared spectra indicates S_a_O_2_ (Fouzas et al 2011). S_p_O_2_ is typically measured using a fingertip clip that passes red and infrared light through the finger and measures light transmission (Fouzas et al 2011).

A Nonin 8500 pulse oximeter (Nonin Medical Inc., Plymouth, MN, USA) was used to measure S_p_O_2_. Probes were selected based on the participant’s weight according to the manufacturer’s recommendations, and attached to the index finger of the left hand, or to the big toe of infants whose fingertip thickness was less than 5mm. Individuals were measured in a calm, resting state (if they were visibly distressed the measurement was not done). As movement can reduce measurement accuracy (Fouzas et al 2011), the child’s hand or foot was held still during the measurement. Insufficient tissue perfusion can also lead to inaccurate readings (Fouzas et al 2011), but results were only recorded when the oximeter’s heart rate indicator showed that there was sufficient blood flow for a reliable measurement. The manufacturer reports accuracy of ±2%, consistent with that for pulse oximetry in general (Jensen et al 1998; Ross and Helms 1990), and the 8500 model is approved for use up to 12,000m altitude and is certified for aeromedical use by the US Air Force.

The normality of distributions for S_p_O_2_ and the anthropometric data were assessed visually using histograms. Anthropometry z score distributions were normal after removing a single strong outlier, and similarly for S_p_O_2_ after excluding two individuals with unusually low readings. Despite previous studies reporting an association between S_p_O_2_ and age (Schult and Canelo-Aybar 2011), no such relationship was found in our data (Pearson’s correlation, p=0.1). Sex differences in S_p_O_2_ were also absent (t-test, p=0.3). Thus age and sex were not included in the analyses, as the anthropometry z scores. The altitude at which the children were studied varied, and S_p_O_2_ decreases with increasing altitude. The sample fell into three natural groups in terms of altitude (Fig. 2), so the data were analysed in three altitude groups and the correlations between altitude and S_p_O_2_ assessed using Spearman’s rank correlation coefficient. Pearson’s correlation was derived between S_p_O_2_ and anthropometry. As other factors varying with altitude, such as socioeconomic status (SES) and healthcare (see Introduction), might also impact on body size and proportions, correlations between altitude of measurement and anthropometry z scores were also conducted to investigate whether the relationships were similar to those between anthropometry and S_p_O_2_, and thus whether a direct effect of S_p_O_2_, rather than other factors associated with altitude, might be inferred. Therefore Spearman’s correlations were derived between altitude group and anthropometry z scores.

Multiple regression analyses of anthropometry z scores on S_p_O_2_ were used to assess their associations including an adjustment for altitude group. To confirm that the use of z scores did not bias the results, the analyses were repeated using raw anthropometry as outcomes, adjusting for sex, age and age^2^ (to cater for nonlinear Analyses were performed using SPSS 21.0 for Windows, with statistical significance defined at p<0.05.

Results

Table 1 summarises the characteristics of the study sample. Summary statistics on stature, sitting height and weight by age group and sex are given in Table S1, and further summary anthropometry can be found in Pomeroy et al. (2012).

The correlation between altitude of measurement and S_p_O_2_ (Fig. 2) is negative, as expected (r=-0.55, p<0.001). Correlations between anthropometry and S_p_O_2_ (Table 2) are significant for ulna, tibia, total upper limb and total lower limb length z scores, but not for stature, head-trunk height, foot or hand length z scores. Overall the correlations are low (≤0.23), indicating weak relationships even where significant.

*** INSERT TABLES 1-2 AND FIGURE 2 HERE ***

Correlations between altitude of measurement and anthropometry z scores rank similarly to those for S_p_O_2_, with those for zeugopod length highest, and those for autopod lengths and head-trunk height low and insignificant (Table 3). Furthermore, these correlations with altitude are generally similar to those with S_p_O_2_ (Table 2). However, none of the anthropometry z scores are significantly related to S_p_O_2_ when adjusted for altitude group (Table 4). The results are confirmed by repeating the analyses using raw anthropometry, which leads to the same conclusions (Tables S2-4).

*** INSERT TABLES 3-4 HERE ***

Discussion

Oxygen saturation and anthropometry z scores are significantly associated in this population of high altitude Andean children. However anthropometry z scores correlate similarly with altitude and S_p_O_2_, and adjusting for altitude the S_p_O_2_  anthropometry associations vanish. This suggests that the relationship between anthropometry and altitude may be partly mediated through S_p_O_2_, which itself is negatively correlated with altitude, but that other factors covarying with altitude also independently influence anthropometry. The low correlations between S_p_O_2_ and anthropometry indicate that, even if the association is causal, factors other than S_p_O_2_ substantially influence anthropometry, in line with evidence that nutritional and other factors, rather than hypoxia, most likely explain the height deficit in highland populations (Greksa 2006).

The results are consistent with previous evidence that total lower limb length is more sensitive to the effects of environmental stress than trunk length (Bogin and Varela-Silva 2010; Frisancho 2007; Gunnell et al 1998; Whitley et al 2008), and that zeugopod lengths are more sensitive to environmental stress than total limb lengths (Meadows Jantz and Jantz 1999; Pomeroy et al 2012). With specific reference to hypoxia, the results agree with those of Bailey and colleagues on Tibetan and Han children, which also showed a stronger association between S_p_O_2_ and absolute or relative tibia length than with total lower limb length or stature (Bailey et al 2007). They are also consistent with a study into the effect of prenatal hypoxia on rats demonstrating reduced total fore- and hindlimb lengths, and that zeugopod lengths were more strongly affected than stylopod (humerus or femur) lengths (Hunter and Clegg 1973). However that study also reported significant reductions in paw length under hypoxic conditions which contrast with our results. Work examining relative tibia, femur and total upper limb lengths in hypoxia-exposed foetuses also suggests that the tibia was shortened relative to the femur, but total upper limb length was unaffected (Lampl et al 2003), again in contrast with our results.

The results suggest that factors other than S_p_O_2_ that covary with altitude may explain much of the altitude-related anthropometric variation. As already outlined, S_p_O_2_ is only one aspect of tissue oxygen delivery, and variation in altitude may have additional hypoxia-related effects on growth that are not captured by S_p_O_2_. S_p_O_2_ also varies diurnally in individuals, for example tending to be lower during sleep (Fouzas et al 2011) and exercise (Brutsaert et al 2000), so the single measurement of S_p_O_2_ recorded in this study may not have captured between-individual variation in S_p_O_2_ that may have had significant impacts on growth. Furthermore, environmental characteristics like SES, temperature and healthcare access decrease with increasing altitude (Leonard et al 1990; Niermeyer et al 2009; Rivera-Ch et al 2008; West et al 2007) while respiratory infection rates increase (Niermeyer et al 2009; Subhi et al 2009), and these may also impact on growth, body size, and body proportions.

Distinguishing between the influences of these different environmental stressors is challenging, since factors such as SES, ambient temperature and tissue oxygenation (the end product of various mechanisms of oxygen delivery at altitude) are hard to characterise. In the present study, participants came from small rural communities where SES is very low and varies little, while diet and access to healthcare are similarly poor. Bailey et al. (2007) reported little variation in SES in their sample of Tibetan and Han children, so argued this could not have accounted for their results, but it remains untested whether even such limited variation could still influence morphology at altitude. Variation in other factors known to influence growth and body size, including maternal phenotype, health and nutrition, and intergenerational effects on maternal and offspring phenotype (Wells 2010) are important considerations for future research. Temperature is unlikely to explain the results, which are inconsistent with the mechanisms thought to link limb growth and temperature (Pomeroy et al 2012). Given the difficulty of separating different influences on morphology at altitude where exposure to multiple stressors is correlated, experimental animal models where other factors can be controlled are likely key to understanding the effects of hypoxia on body size and proportions.

In terms of underlying mechanisms, our results are inconsistent with a proximo-distal decrease in available resources along the limb, as suggested in Lampl’s distal blood flow model (Lampl et al 2003). This hypothesis states that foetuses exposed to hypoxia due to maternal diabetes or smoking show reduced tibia length, but not reduced total upper limb or femur lengths, due to the nature of the foetal circulation and the diminution of blood oxygen availability with distance from the placenta, reaching its lowest levels in the distal lower limb (tibia). The model implies that hands and feet are most affected by hypoxia, though this was not investigated by Lampl et al. (2003). However this was not the pattern observed here.

The results are more consistent with the thrifty phenotype hypothesis as applied to limb lengths (Pomeroy et al 2012). The thrifty phenotype hypothesis (Hales and Barker 1992) states that when resources are limited, growth is prioritised in organs or parts of the body where function would be most compromised by inadequate growth. In comparing children from the Peruvian highlands (including the sample from the present study) with those from the Peruvian lowlands who experience markedly lower levels of environmental stress in terms of socioeconomic factors, healthcare access, hypoxia and cold exposure, we documented a similar pattern (Pomeroy et al 2012). Zeugopod lengths showed the greatest differences between populations, followed by total limb lengths, while differences were smaller in hand and foot lengths and smallest in head-trunk height. The trunk may be relatively protected as it houses the major organs, while autopod lengths may also be protected due to their critical roles in manipulation and substrate interaction during locomotion. While this suggestion has not been demonstrated empirically, it has been proposed that greater canalisation of autopod size compared with the stylopod and zeugopod may be explained in this way (Rolian 2008; Rolian 2009; Young and Hallgrímsson 2005). Our analyses suggest that S_p_O_2_ represents one altitude-associated signal of supply, to which the limb components respond in a hierarchical manner.

In terms of proximate mechanisms, trunk size may be maintained at the expense of limb lengths through peripheral vasoconstriction which may reduce nutrient delivery to the limbs, a response observed in humans and animal models exposed to hypoxia or nutritional stress (Burrage et al 2009; Celander 1960; Edelstone and Rudolph 1979; Gardner et al 2002; Giussani et al 1994; Giussani et al 2005; Hawkins et al 2000; Kidd et al 1966; Krampl et al 2001; Llanos et al 2007; Morrison 2008; Mulder et al 1998; Powers and Swyer 1977; Rudolph 1984; Williams et al 2005). Existing studies of regional blood flow generally measure only lower limb blood supply, and data on upper limb circulation are lacking, as are direct studies of blood flow in relation to limb segment lengths in animals or humans. Studies of limb segment blood flow demonstrate proportionally greater flow (corrected for element size) to the stylopod than the zeugopod, although there is evidence for variation with age (proximo-distal gradients decrease with age) and among species (Morris and Kelly 1980; Nakano et al 1986; Tothill et al 1985; Tothill and MacPherson 1986). In addition, it is well accepted that blood supply affects limb growth (Brashear 1963; Brodin 1955; Serrat 2007; Tomita et al 1986).

A strength of the present study is the predominance of infants and young children in the sample, since plasticity is thought to be greatest at younger ages (Lucas 1991; Mei et al 2004; Smith et al 1976) and thus patterns of body size and proportion in relation to S_p_O_2_ are potentially strongest in such individuals. While foetal growth may be the most plastic of all, prenatal environmental influences are mediated through maternal phenotype (Wells 2003). Limitations of this study include its short timescale, so it is not possible to test whether the patterns maintain into adulthood, or to investigate the interaction between hypoxia and nutritional status on growth suggested by Bailey et al. (2007). It also remains to be demonstrated how other aspects of oxygen delivery relate to body proportions and growth at altitude. Nonetheless the study has important implications for public health policy. As correlations between S_p_O_2_ and anthropometry were low, even where significant, this implies that other factors exert much greater influences on growth and variation in body size and proportions, likely nutrition and healthcare as others have argued previously (e.g. Greksa 2006). Therefore interventions that serve to improve nutrition and health in highland communities are likely to be effective in improving growth.

In conclusion, this study demonstrates significant correlations between S_p_O_2_ or altitude and limb and limb segment length z scores among Andean children. The results indicate that associations are strongest with zeugopod length and then total limb length z scores, but weaker and insignificant with head-trunk height and autopod length z scores. Part of the association between altitude and limb measurement z scores seems to be mediated by S_p_O_2_, although correlations are relatively weak, and other factors that covary with altitude are likely to play a major role in influencing body size and proportions. Future work should aim to explore further the effects of hypoxia on growth and body proportions to elucidate the details of the underlying mechanisms, and to distinguish the effects of hypoxia and other environmental stress exposures at altitude.

**Acknowledgements**

Thank you to Lilia Cabrera and Angela Huamán Gómez of PRISMA, and all the field staff for their assistance in participant recruitment and data collection. Thanks to Dr. Antonio Bernabe Ortiz for assistance with establishing the project, and to Rie Goto for advice. We are also grateful to the Ayacucho Department Health Directorate (DIRESA) for their support, and especially to all the participants and their families who generously gave their time to take part in the study. Thanks also to the editor and two anonymous reviewers whose comments helped to improve the manuscript.

**Literature cited**

Bailey SM, Hu XM. 2002. High-altitude growth differences among Chinese and Tibetan children. In: Gilli G, Schell LM, Benso L, editors. Human Growth from Conception to Maturity. London: Smith-Gordon. p 237-247.

Bailey SM, Xu J, Feng JH, Hu X, Zhang C, Qui S. 2007. Tradeoffs between oxygen and energy in tibial growth at high altitude. Am J Hum Biol 19(5):662-8.

Baker PT. 1976. Evolution of a project: theory, method and sampling. Man in the Andes: A Multidisciplinary Study of High Altitude Quechua. Stroudsburg, Pennsylvania: Dowden, Hutchinson and Ross, Inc. p 1-20.

Beall CM. 1981. Optimal birthweights in Peruvian populations at high and low altitudes. Am J Phys Anthropol 56(3):209-216.

Beall CM. 2001. Adaptations to altitude: a current assessment. Annu Rev Anthropol 30(1):423-456.

Beall CM. 2007. Two routes to functional adaptation: Tibetan and Andean high-altitude natives. Proc Natl Acad Sci U S A 104(Suppl 1):8655-8660.

Beall CM, Blangero J, Williams-Blangero S, Goldstein MC. 1994. Major gene for percent of oxygen saturation of arterial hemoglobin in Tibetan highlanders. Am J Phys Anthropol 95(3):271-6.

Beall CM, Laskowski D, Strohl KP, Soria R, Villena M, Vargas E, Alarcon AM, Gonzales C, Erzurum SC. 2001. Pulmonary nitric oxide in mountain dwellers. Nature 414(6862):411-412.

Beall CM, Song K, Elston RC, Goldstein MC. 2004. Higher offspring survival among Tibetan women with high oxygen saturation genotypes residing at 4,000 m. Proc Natl Acad Sci U S A 101(39):14300-14304.

Beall CM, Strohl KP, Blangero J, Williams-Blangero S, Decker MJ, Brittenham GM, Goldstein MC. 1997. Quantitative genetic analysis of arterial oxygen saturation in Tibetan highlanders. Hum Biol 69(5):597-604.

Bennett A, Sain SR, Vargas E, Moore LG. 2008. Evidence that parent-of-origin affects birth-weight reductions at high altitude. Am J Hum Biol 20(5):592-7.

Bogin B, Smith P, Orden AB, Varela Silva MI, Loucky J. 2002. Rapid change in height and body proportions of Maya American children. Am J Hum Biol 14(6):753-61.

Bogin B, Varela-Silva MI. 2010. Leg length, body proportion, and health: a review with a note on beauty. Int J Environ Res Public Health 7(3):1047-1075.

Brashear HR, Jr. 1963. Epiphyseal avascular necrosis and its relation to longitudinal bone growth. J Bone Joint Surg Am 45:1423-38.

Brodin H. 1955. Longitudinal bone growth, the nutrition of the epiphyseal cartilages and the local blood supply; an experimental study in the rabbit. Acta Orthop Scand Supplementum 20:1-92.

Brutsaert TD, Araoz M, Soria R, Spielvogel H, Haas JD. 2000. Higher arterial oxygen saturation during submaximal exercise in Bolivian Aymara compared to European sojourners and Europeans born and raised at high altitude. Am J Phys Anthropol 113: 169-181.

Burrage DM, Braddick L, Cleal JK, Costello P, Noakes DE, Hanson MA, Green LR. 2009. The late gestation fetal cardiovascular response to hypoglycaemia is modified by prior peri-implantation undernutrition in sheep. J Physiol 587(3):611-624.

Celander O. 1960. Blood flow in the foot and calf of the newborn. A plethysmographic study. Acta Pædiatr 49(4):488-496.

Cole TJ. 1990. The LMS method for constructing normalized growth standards. Eur J Clin Nutr 44(1):45-60.

Cole TJ, Green PJ. 1992. Smoothing reference centile curves: The LMS method and penalized likelihood. Stat Med 11(10):1305-1319.

Edelstone DI, Rudolph AM. 1979. Preferential streaming of ductus venosus blood to the brain and heart in fetal lambs. Am J Physiol Heart Circ Physiol 237(6):H724-H729.

Erzurum SC, Ghosh S, Janocha AJ, Xu W, Bauer S, Bryan NS, Tejero J, Hemann C, Hille R, Stuehr DJ and others. 2007. Higher blood flow and circulating NO products offset high-altitude hypoxia among Tibetans. Proc Natl Acad Sci U S A 104(45):17593-17598.

Fouzas S, Priftis KN, Anthracopoulos MB. 2011. Pulse oximetry in pediatric practice. Pediatrics 128(4):740-752.

Frisancho AR. 2007. Relative leg length as a biological marker to trace the developmental history of individuals and populations: growth delay and increased body fat. Am J Hum Biol 19(5):703-10.

Gardner DS, Fletcher AJW, Bloomfield MR, Fowden AL, Giussani DA. 2002. Effects of prevailing hypoxaemia, acidaemia or hypoglycaemia upon the cardiovascular, endocrine and metabolic responses to acute hypoxaemia in the ovine fetus. J Physiol 540(1):351-366.

Giussani DA, Phillips PS, Anstee S, Barker DJ. 2001. Effects of altitude versus economic status on birth weight and body shape at birth. Pediatr Res 49(4):490-4.

Giussani DA, Spencer JAD, Hanson MA. 1994. Fetal cardiovascular reflex responses to hypoxaemia. Fetal Matern Med Rev 6(01):17-37.

Giussani DA, Thakor AS, Frulio R, Gazzolo D. 2005. Acute hypoxia increases S100β protein in association with blood flow redistribution away from peripheral circulations in fetal sheep. Pediatr Res 58(2):179-184.

Greksa LP. 2006. Growth and development of Andean high altitude residents. High Alt Med Biol 7(2):116-24.

Gunnell DJ, Smith GD, Frankel SJ, Kemp M, Peters TJ. 1998. Socio-economic and dietary influences on leg length and trunk length in childhood: a reanalysis of the Carnegie (Boyd Orr) survey of diet and health in prewar Britain (1937-39). Paediatr Perinat Epidemiol 12 (Suppl 1):96-113.

Haas JD, Baker PT, Hunt EE, Jr. 1977. The effects of high altitude on body size and composition of the newborn infant in Southern Peru. Hum Biol 49(4):611-28.

Hales CN, Barker DJ. 1992. Type 2 (non-insulin-dependent) diabetes mellitus: the thrifty phenotype hypothesis. Diabetologia 35(7):595-601.

Hawkins P, Steyn C, McGarrigle HHG, Calder NA, Saito T, Stratford LL, Noakes DE, Hanson MA. 2000. Cardiovascular and hypothalamic-pituitary-adrenal axis development in late gestation fetal sheep and young lambs following modest maternal nutrient restriction in early gestation. Reprod Fertil Dev 12(8):443-464.

Hunter C, Clegg EJ. 1973. Changes in skeletal proportions of the rat in response to hypoxic stress. J Anat 114(2):201-19.

Jensen LA, Onyskiw JE, Prasad NGN. 1998. Meta-analysis of arterial oxygen saturation monitoring by pulse oximetry in adults. Heart & Lung: The Journal of Acute and Critical Care 27(6):387-408.

Julian CG, Hageman JL, Wilson MJ, Vargas E, Moore LG. 2011. Lowland origin women raised at high altitude are not protected against lower uteroplacental O2 delivery during pregnancy or reduced birth weight. Am J Hum Biol 23(4):509-516.

Julian CG, Vargas E, Armaza JF, Wilson MJ, Niermeyer S, Moore LG. 2007. High-altitude ancestry protects against hypoxia-associated reductions in fetal growth. Arch Dis Child Fetal Neonatal Ed 92(5):F372-7.

Julian CG, Wilson MJ, Lopez M, Yamashiro H, Tellez W, Rodriguez A, Bigham AW, Shriver MD, Rodriguez C, Vargas E and others. 2009. Augmented uterine artery blood flow and oxygen delivery protect Andeans from altitude-associated reductions in fetal growth. Am J Physiol Regul Integr Comp Physiol 296(5):R1564-75.

Kidd L, Levison H, Gemmel P, Aharon A, Swyer PR. 1966. Limb blood flow in the normal and sick newborn: a plethysmographic study. Am J Dis Child 112(5):402-407.

Krampl E, Chalubinski K, Schatten C, Husslein P. 2001. Does acute hypoxia cause fetal arterial blood flow redistribution? Ultrasound Obstet Gynecol 18(2):175-7.

Lampl M, Kuzawa CW, Jeanty P. 2003. Prenatal smoke exposure alters growth in limb proportions and head shape in the midgestation human fetus. Am J Hum Biol 15(4):533-46.

Leonard WR, Leatherman TL, Carey JW, Thomas RB. 1990. Contributions of nutrition vs. hypoxia to growth in rural Andean populations. Am J Hum Biol 2:613-626.

Llanos AJ, Riquelme RA, Herrera EA, Ebensperger G, Krause B, Reyes RV, Sanhueza EM, Pulgar VM, Behn C, Cabello G and others. 2007. Evolving in thin air - Lessons from the llama fetus in the altiplano. Respir Physiol Neurobiol 158(2-3):298-306.

Lucas A. 1991. Programming by early nutrition in man. In: Bock GR, Whelan J, editors. The Childhood Environment and Adult Disease. Chichester: John Wiley and Sons Ltd. p 38-55.

McClung J. 1969. Effects of High Altitude on Human Birth: Observations on Mothers, Placentas, and the Newborn in Two Peruvian Populations. Cambridge, MA: Harvard University Press.

Meadows Jantz L, Jantz RL. 1999. Secular change in long bone length and proportion in the United States, 1800-1970. Am J Phys Anthropol 110(1):57-67.

Mei Z, Grummer-Strawn LM, Thompson D, Dietz WH. 2004. Shifts in Percentiles of Growth During Early Childhood: Analysis of Longitudinal Data From the California Child Health and Development Study. Pediatrics 113(6):e617-627.

Moore LG. 2003. Fetal growth restriction and maternal oxygen transport during high altitude pregnancy. High Alt Med Biol 4(2):141-56.

Moore LG, Niermeyer S, Zamudio S. 1998. Human adaptation to high altitude: regional and life-cycle perspectives. Yrbk Phys Anthropol 27:25-64.

Morris M, Kelly P. 1980. Use of tracer microspheres to measure bone blood flow in conscious dogs. Calcif Tissue Int 32(1):69-76.

Morrison JL. 2008. Sheep models of intauterine growth restriction: fetal adaptations and consequences. Clin Exp Pharmacol Physiol 35(7):730-743.

Mulder ALM, van Golde JC, Prinzen FW, Blanco CE. 1998. Cardiac output distribution in response to hypoxia in the chick embryo in the second half of the incubation time. J Physiol 508(1):281-287.

Nakano T, Thompson JR, Christopherson RJ, Aherne FX. 1986. Blood flow distribution in hind limb bones and joint cartilage from young growing pigs. Can J Vet Res 50(1):96-100.

Niermeyer S, Andrade Mollinedo P, Huicho L. 2009. Child health and living at high altitude. Arch Dis Child 94(10):806-811.

Pan H, Cole TJ. 2010. LMSchartmaker, a program to construct growth references using the LMS method. Version 2.43. http://www.healthforallchildren.co.uk/.

Pomeroy E, Stock JT, Stanojevic S, Miranda JJ, Cole TJ, Wells JCK. 2012. Trade-Offs in Relative Limb Length among Peruvian Children: Extending the Thrifty Phenotype Hypothesis to Limb Proportions. PLoS One 7(12):e51795.

Powers WF, Swyer PR. 1977. Diminished limb blood flow in infants with transposition of the great vessels: an adaptation to chronic hypoxia? Acta Paediatr 66(2):205-209.

Rivera-Ch M, Castillo A, Huicho L. 2008. Hypoxia and other environmental factors at high altitude. Int J Environ Health 2(1):92-106.

Rolian C. 2008. Developmental basis of limb length in rodents: evidence for multiple divisions of labor in mechanisms of endochondral bone growth. Evol Dev 10(1):15-28.

Rolian C. 2009. Integration and evolvability in primate hands and feet. Evol Biol 36(1):100-117.

Ross RI, Helms PJ. 1990. Comparative accuracy of pulse oximetry and transcutaneous oxygen in assessing arterial saturation in pediatric intensive care. Crit Care Med 18(7):725-727.

Rudolph AM. 1984. The fetal circulation and its response to stress. J Dev Physiol 6(1):11-9.

Samaras TT, editor. 2007. Human Body Size and the Laws of Scaling: Physiological, Performance, Growth, Longevity and Ecological Ramifications. New York: Nova Science Publishers Inc.

Schult S, Canelo-Aybar C. 2011. Oxygen saturation in healthy children aged 5 to 16 years residing in Huayllay, Peru at 4340 m. High Alt Med Biol 12(1):89-92.

Serrat MA. 2007. Experimentally-determined Tissue Temperature Modulates Extremity Growth in Mammals: A Potential Comprehensive Explanation of Allen's Rule. Unpublished PhD Thesis: Kent State University.

Smith DW, Truog W, Rogers JE, Greitzer LJ, Skinner AL, McCann JJ, Sedgwick Harvey MA. 1976. Shifting linear growth during infancy: Illustration of genetic factors in growth from fetal life through infancy. J Pediatr 89(2):225-230.

Stinson S. 2009. Nutritional, developmental, and genetic influences on relative sitting height at high altitude. Am J Hum Biol 21(5):606-613.

Subhi R, Smith K, Duke T. 2009. When should oxygen be given to children at high altitude? A systematic review to define altitude-specific hypoxaemia. Arch Dis Child 94(1):6-10.

Tomita Y, Tsai TM, Steyers C, Ogden L, Jupiter JB, Kutz JE. 1986. The role of the epiphyseal and metaphyseal circulations on longitudinal growth in the dog: an experimental study. J Hand Surg Am 11(3):375-82.

Tothill P, Hooper G, McCarthy I, Hughes S. 1985. The variation with flow-rate of the extraction of bone-seeking tracers in recirculation experiments. Calcif Tissue Int 37(3):312-317.

Tothill P, MacPherson JN. 1986. The distribution of blood flow to the whole skeleton in dogs, rabbits and rats measured with microspheres. Clin Phys Physiol Meas 7(2):117-23.

Unger C, Weiser JK, McCullough RE, Keefer S, Moore LG. 1988. Altitude, low birth weight, and infant mortality in Colorado. JAMA 259(23):3427-3432.

Wells JC. 2003. The thrifty phenotype hypothesis: thrifty offspring or thrifty mother? J Theor Biol 221(1):143-61.

Wells JCK. 2010. Maternal capital and the metabolic ghetto: An evolutionary perspective on the transgenerational basis of health inequalities. Am J Hum Biol 22(1):1-17.

West JB, Schoene RB, Milledge JS, Ward MP. 2007. High Altitude Medicine and Physiology. London: Hodder Arnold.

Whitley E, Gunnell D, Davey Smith G, Holly JMP, Martin RM. 2008. Childhood circumstances and anthropometry: The Boyd Orr cohort. Ann Hum Biol 35(5):518-534.

Williams SJ, Campbell ME, McMillen IC, Davidge ST. 2005. Differential effects of maternal hypoxia or nutrient restriction on carotid and femoral vascular function in neonatal rats. Am J Physiol Regul Integr Comp Physiol 288(2):R360-R367.

World Medical Association. 2008. WMA Declaration of Helsinki - Ethical Principles for Medical Research Involving Human Subjects. World Medical Association: http://www.wma.net/en/30publications/10policies/b3/ accessed 03/05/2011.

Young NM, Hallgrímsson B. 2005. Serial homology and the evolution of mammalian limb covariation structure. Evolution 59(12):2691-704.

Zamudio S, Moore LG. 2000. Altitude and fetal growth: current knowledge and future directions. Ultrasound Obstet Gynecol 16(1):6-8.

**Tables**

**Table 1. Summary statistics of the study sample**

| **Variable** | **Statistic** | **Value** |
| --- | --- | --- |
| Sample size | n (males, females) | 165 (82, 83) |
| Altitude of measurement (m) | Median | 3564 |
|  | Interquartile range | 3306-3823 |
|  | Range | 3150-4415 |
| S_p_O_2_ (%) | Median | 90 |
|  | Interquartile range | 88-92 |
|  | Range | 81-99 |
| Age (years) | Mean | 5.25 |
|  | Standard deviation | 3.59 |
|  | Range | 0.5-14.4 |

**Table 2.** **Correlations between S_p_O_2_ and anthropometry z scores**

| **Outcome z score** | **Pearson r** | **p** | **n** |
| --- | --- | --- | --- |
| Ulna length | 0.23 | **0.003** | 163 |
| Tibia length | 0.22 | **0.004** | 164 |
| Total upper limb length | 0.19 | **0.02** | 159 |
| Total lower limb length | 0.17 | **0.03** | 165 |
| Stature | 0.15 | 0.06 | 165 |
| Head-trunk height | 0.08 | 0.3 | 165 |
| Foot length | 0.07 | 0.4 | 164 |
| Hand length | 0.05 | 0.5 | 156 |

**Bold** indicates significant p values

**Table 3. Correlations between altitude and anthropometry z scores**

| **Outcome z score** | **Spearman r** | **p** | **n** |
| --- | --- | --- | --- |
| Tibia length | -0.28 | **<0.001** | 164 |
| Ulna length | -0.26 | **0.001** | 163 |
| Total lower limb length | -0.3 | **0.001** | 165 |
| Total upper limb length | -0.18 | **0.03** | 159 |
| Stature | -0.1 | 0.07 | 165 |
| Hand length | -0.08 | 0.3 | 156 |
| Foot length | -0.04 | 0.6 | 164 |
| Head-trunk height | 0.006 | 0.9 | 165 |

**Bold** indicates significant p values

**Table 4. Results of multiple regression models of anthropometry z scores on S_p_O_2_ and altitude**

| **Outcome z score** | **3,700-4,000m altitude^. a^** | | | **4,200-4,500m altitude** | | | **S_p_O_2_** | | |
| --- | --- | --- | --- | --- | --- | --- | --- | --- | --- |
|  | **B** | **SE** | **p** | **B** | **SE** | **p** | **B** | **SE** | **p** |
| Tibia length | -0.32 | 0.11 | **0.004** | -0.67 | 0.19 | **0.001** | 0.007 | 0.018 | 0.7 |
| Total lower limb length | -0.37 | 0.12 | **0.004** | -0.71 | 0.22 | **0.002** | -0.002 | 0.021 | 0.9 |
| Ulna length | -0.24 | 0.10 | **0.02** | -0.43 | 0.18 | **0.02** | 0.018 | 0.017 | 0.3 |
| Total upper limb length | -0.22 | 0.11 | **0.05** | -0.12 | 0.19 | 0.5 | 0.027 | 0.018 | 0.1 |
| Stature | -0.13 | 0.12 | 0.3 | -0.42 | 0.22 | 0.06 | 0.009 | 0.021 | 0.6 |
| Foot length | -0.00 | 0.13 | 0.9 | -0.20 | 0.22 | 0.4 | 0.004 | 0.021 | 0.8 |
| Hand length | -0.02 | 0.13 | 0.9 | -0.04 | 0.22 | 0.8 | 0.009 | 0.021 | 0.7 |
| Head-trunk height | 0.13 | 0.13 | 0.3 | 0.04 | 0.23 | 0.8 | 0.021 | 0.022 | 0.3 |

**Bold** indicates significant p values

**^a^** 2 dummy variables for altitude group with lowest altitude group (3,150-3,400m) as the reference group

**Figure legends**

Fig. 1. Map showing location of study sites in the central Peruvian highlands.

Fig. 2. Scatterplot of arterial oxygen saturation (S_p_O_2_) against altitude where measurements were taken, demonstrating the expected decrease in S_p_O_2_ with increasing altitude.
